# Setting the Standard: Developing a High-Consequence Infectious Disease Preparedness Policy for a Comprehensive Cancer Hospital

**DOI:** 10.1017/ash.2025.403

**Published:** 2025-09-24

**Authors:** Adina Feldman, Sherry Cantu, Rebecca Poths, Jane Powell, Guy Handley, Amy Spallone

**Affiliations:** 1 MD Anderson Cancer Center; 2 MD Anderson Cancer Center; 3 Univ of Texas MD Anderson Cancer Center; 4 MD Anderson Cancer Center; 5 MD Anderson Cancer Center; 6 MD Anderson Cancer Center

## Abstract

**Background:** In 2024, The Joint Commission (TJC) introduced Standard IC.07.01.01 to bolster hospitals’ preparedness for high-consequence infectious diseases (HCIDs) such as novel Influenza, Ebola, and SARS-CoV-2. The standard emphasizes the need for an effective framework for managing emerging pathogens. While a definitive list of HCIDs does not exist, TJC defines HCIDs as, “novel or reemerging infectious agents characterized by high transmissibility, limited or no medical countermeasures, high mortality, and a need for rapid identification and stringent infection control.” We outline the process of developing a policy at a National Cancer Institute-designated cancer center to ensure prompt and efficient management of suspected HCID cases. **Methods:** The policy development process began with a thorough review of existing hospital policies, infection control protocols, and environmental safety guidelines. Stakeholders from multiple departments including Environmental Health and Safety, Facilities Management, Employee Health and Campus police were consulted in the policy development process. A needs assessment was followed to identify gaps and areas requiring improvement. The policy was designed using key resources, including the Centers for Disease Control and Prevention (CDC), the World Health Organization (WHO), and the National Emerging Special Pathogens Training and Education Center (NETEC). A framework based on the CDC’s “Identify, Inform, and Isolate” model was employed, with tailored procedures addressing both clinical and operational aspects of preparedness and response. **Results:** The final policy grouped HCIDs into five categories: (1) Viral Hemorrhagic Fevers (VHF), (2) Novel Respiratory Viruses, (3) Measles, (4) Bioterrorism Agents, and (5) Other Emerging Pathogens. For each category, the policy delineated specific identification criteria, isolation protocols, and management procedures. It also provided guidance on engineering controls, visitor management, patient placement, environmental cleaning, and transportation. Additionally, the policy included external resources on clinical treatments and broader infection control issues. **Conclusion:** The development of an HCID policy in accordance with TJC Standard IC.07.01.01 provides a robust framework for hospital preparedness in managing high-consequence infectious diseases. By collaborating with Infection Control teams, healthcare institutions can develop protocols that enable swift, effective responses to emerging pathogens, ensuring adaptability during outbreaks. This policy not only facilitates TJC compliance but also enhances hospital readiness for future infectious disease threats. Moreover, it offers a replicable model that can assist other healthcare organizations in strengthening their emergency response capabilities and maintaining survey readiness in an evolving healthcare landscape.

